# Scutellaria baicalensis georgi is a promising candidate for the treatment of autoimmune diseases

**DOI:** 10.3389/fphar.2022.946030

**Published:** 2022-09-16

**Authors:** Jun Wang, Shanshan Chen, Jizhou Zhang, Jiasi Wu

**Affiliations:** ^1^ Department of Pharmacy and Emergency, Yaan People’s Hospital, Yaan, PR, China; ^2^ Acupuncture and Tuina School, Chengdu University of Traditional Chinese Medicine, Chengdu, PR, China

**Keywords:** scutellaria baicalensis, autoimmune diseases, inflammatory response, inflammasome, inflammatory injury

## Abstract

Autoimmune diseases a group of disorders elicited by unexpected outcome of lymphocytes self-tolerance failure, and the common members of which include multiple sclerosis, systemic lupus erythematosus, inflammatory bowel disease, rheumatoid arthritis, and type 1 diabetes mellitus, etc. The pathogenesis of autoimmune diseases is not fully understood and the current therapeutic regimen’s inefficacy in certain cases coupled with low rates of success, exorbitant financial burden, as well as numerous side effects, which do open new avenues for the role of natural products as novel therapeutic agents for auto-inflammatory disorders. Scutellaria baicalensis Georgi is a well-known and widely-recognized herbal medicine with certain ameliorative effect on diverse inflammation-involved dysfunction. Though recent advances do highlight its potential to be applied in the fight against autoimmune diseases, the specific mechanism and the related opinion on the exploring possibility are still limited which hampered the further progress. Here in this timeline review, we traced and collected the evidence of how Scutellaria baicalensis Georgi and its bioactive contents, namely baicalin, baicalein, wogonoside and wogonin affect autoimmune diseases. Moreover, we also discussed the clinical implications and therapeutic potential of Scutellaria baicalensis Georgi and its bioactive contents in autoimmune diseases treatment.

## Introduction

Immune system is generally noted as a double-edged sword due to its nature of either heal or even harm the physiological mechanism of human beings. Defined as dysfunction of antigenic recognition and immune cells elimination, autoimmune diseases (ADS) are undesired consequence caused by self-tolerance failure of lymphocytes, the chief drive of their development (Rosetti et al., 2022). By far, a series of ADS, either systemic or organ-specific, have been identified and described. The typical ones include multiple sclerosis, systemic lupus erythematosus (SLE), inflammatory bowel disease (IBD), rheumatoid arthritis (RA), type 1 diabetes mellitus (T1DM), autoimmune hepatitis and autoimmune orchitis (Wu et al., 2021), details shown in Table 1. Epidemiological data indicated ADS afflict approximately 10% of the population worldwide, the percentage increase of which was 3.7, 6.2, 6.3 and 7.1% for neurological, gastrointestinal, endocrinological and rheumatic autoimmune diseases, respectively (Rengasamy et al., 2019). By far, the underlying pathogenesis mechanism of ADS are not fully unveiled though it may be closely related to abnormal immune modulation, internal and external environmental factor (Boardman and Levings, 2022). In fact, the current therapeutic criteria for ADS includes janus kinase (JAK) inhibitors and several specific monoclonal antibodies which is somehow based on the manifestations that complemented with laboratory test (Tavakolpour, 2017; Wu et al., 2018; Tanaka et al., 2022). With a goal of introducing a new generation of treating regimen with fewer life-threatening side effect, there’s an urgent need to explore novel small-molecule alternative medicine from natural resources.

**TABLE 1:** Typical systemic or organ-specific ADS (Wu et al., 2021).

Targeting organs	ADs	Targeting organs	ADs
Brain and spinal cord	multiple sclerosis	Skin and hair	psoriasis vulgaris
	narcolepsy		SLE
	optical neuromyelitis		alopecia areata
Digestive system	IBD		pemphigus vulgaris
	celiac disease		vitiligo
	autoimmune gastritis	Joints	RA
	primary biliary cholangitis		psoriatic arthritis
	autoimmune hepatitis		axial spondylarthritis
Endocrine glands	T1DM	Muscle	myasthenia gavis
Reproductive organs	autoimmune orchitis		polymyositis
Kidney	Lupus nephritis	Ophthalmology	EAU

**FIGURE 1:**
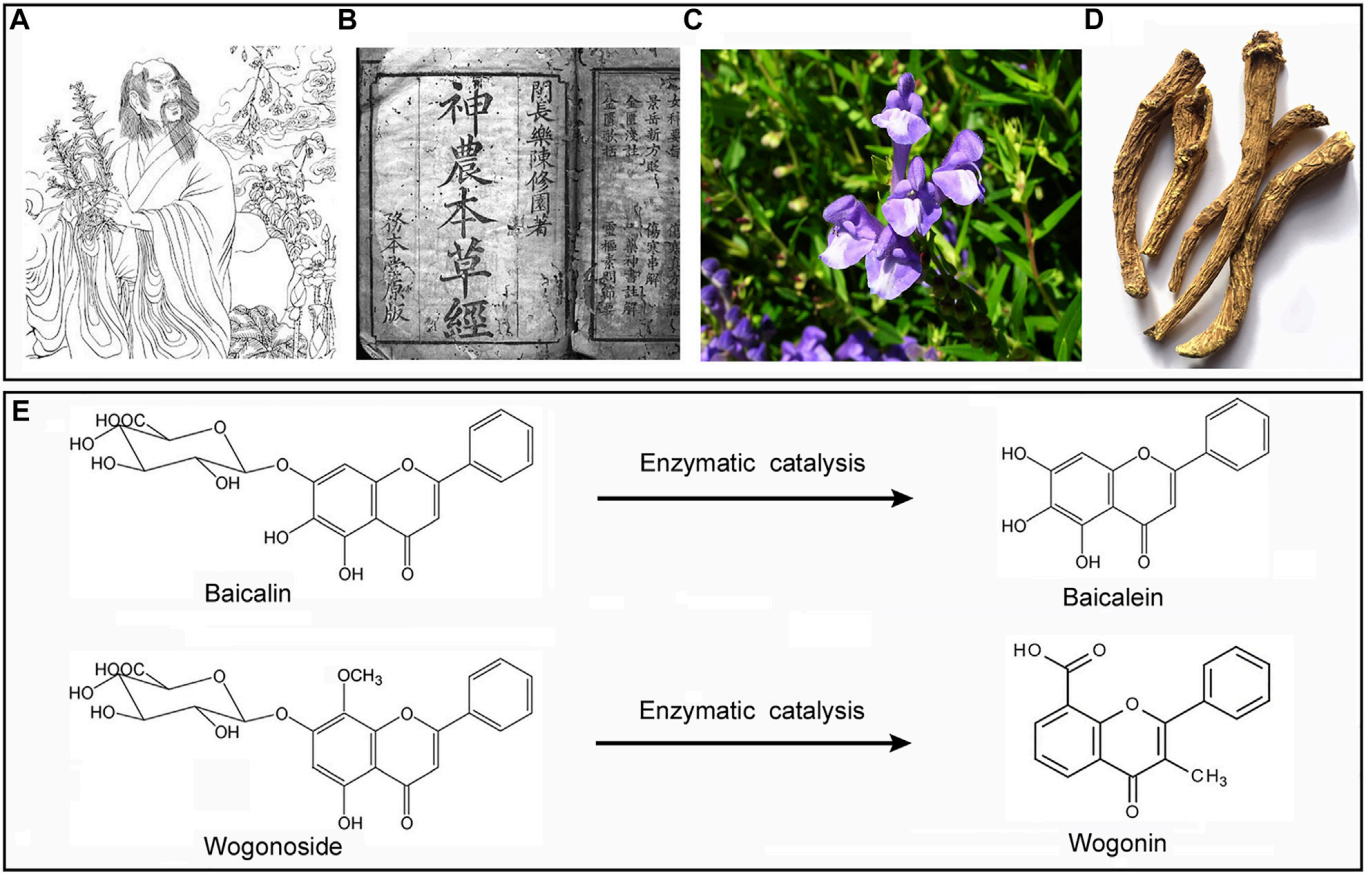
SBG was originally documented in Shennong’s **(A)** Shennong materia Medica **(B)**. The plant **(C)**, dry root **(D)**, and the chemical structure of WGS/WGI **(E)**.

Baicalin (BAI)and its aglycone baicalein (BAE), as well as wogonin (WGI) and its aglycone wogonoside (WGS) are bioactive flavonoids which can be extracted from a well-known Chinses herb named Scutellaria baicalensis Georgi (SBG) originally documented in Shennong Bencao Jing (also known as the Divine Farmer’s Materia Medica) which was written between 200 and 250 AD (depicted in Figure 1). Another ancient medical book named Bencao Gangu (also known as the authoritative Materia Medica, accomplished in 1953) described SGB’s outstanding pharmacological properties against a range of disorders in details, and the author, Lishizhen, self-administrated SBG and reported it was cure for severe lung infection (Zhao et al., 2016). SBG was a representative medicinal plant with natural property of fire-purging and toxin-removing, which was attributed to its flavonoid contents that mentioned above. Clinically, when used in combination therapy, SBG demonstrated certain effect in treatment of non-small cell pulmonary carcinomas. Besides, modern evidence showed SGB potentiated positive outcome when applied to treat inflammatory disorders, respiratory tract infections, diarrhea, dysentery, liver dysfunctions, hypertension, hemorrhaging, as well as insomnia (Li-Weber, 2009). By far, systemic review which focused on the interaction between SBG and ADS is yet limited. On the other hand, several reviews concerning SBG’s small molecular contents have been released during the past decades, in which the general therapeutic and pharmacological effect (Huynh et al., 2020), prevention against cancer (Butt et al., 2021; Banik et al., 2022), controlling of diabetic cardiomyopathy (Khan and Kamal, 2019), protection against ischemia-induced neurodegeneration (Pan et al., 2021) as well as amelioration on ocular disorders (Xiao et al., 2014) of them were analyzed and discussed, while there’s no consensus on whether SBG and its compounds were promising candidates being capable of blocking ADS pathogenesis progress. Therefore, here in this timeline, we will concern about the virtue of SBG from the viewpoint of both *in vivo* and *in vitro* correlated with ADS immunologic pathologies, and meanwhile put forward synoptic outlook regarding these bioactive small-molecular compounds in SBG.

## Scutellaria baicalensis georgi and its flavonoid compounds are promising candidates to restrain autoimmune diseases pathogenesis via inactivating inflammatory responses

**FIGURE 2:**
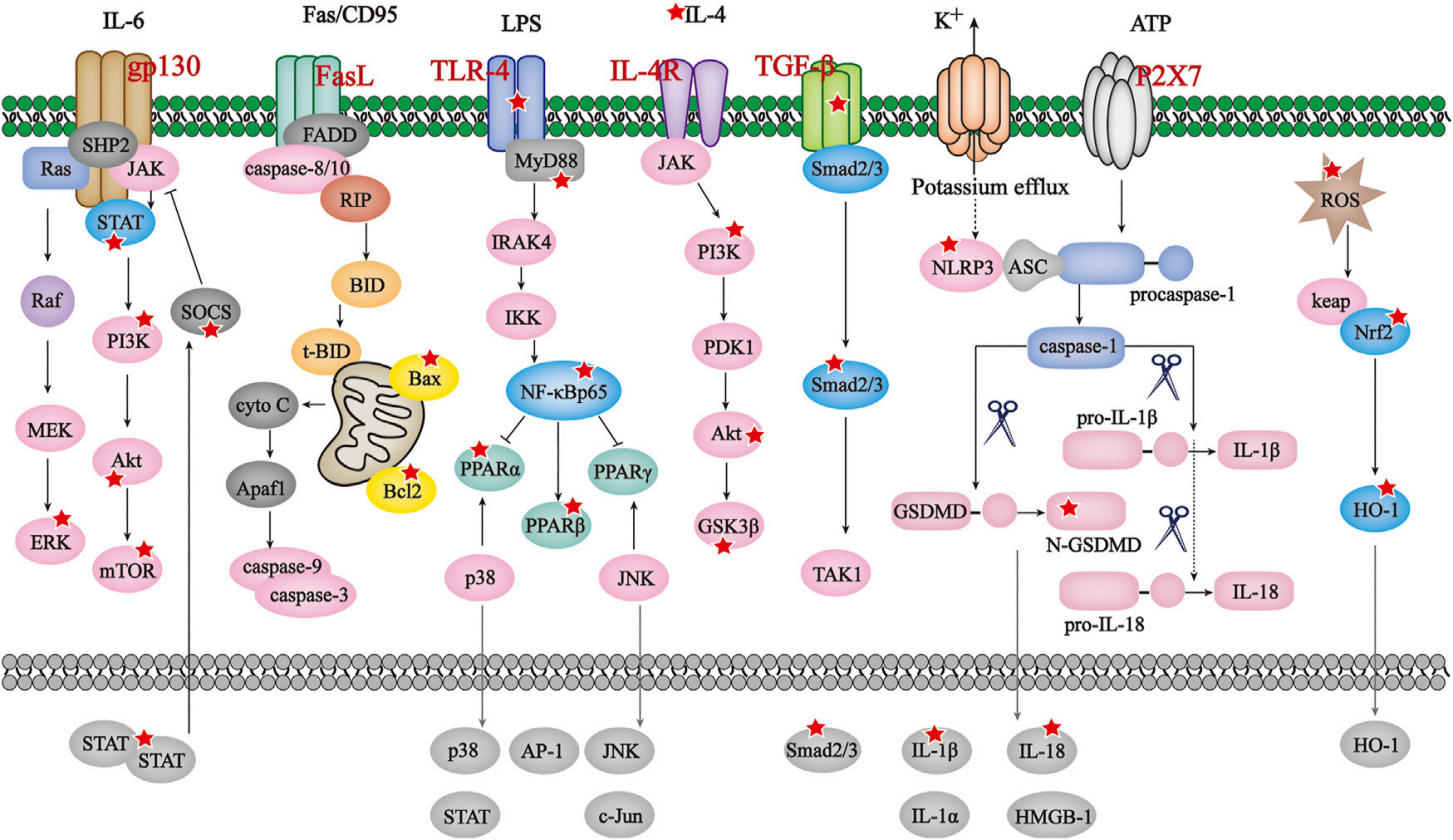
The principal pathways modulated by SBG and its compounds in ADS. JAK/STAT/mTOR, BAX/Bcl2/caspase-3/9, TLR4/NF-κB/PPAR, IL-4/PI3K/Akt/GSK3β, TGF-β/Smad2/3,NLRP3/caspase-1/GSDMD and ROS/Nrf2/HO-1 pathways are the principal signaling modulated by SBG and its compounds in ADS. The reported molecules regulated by SBG are marked with a red star.

As mentioned in previous chapters, aberrant immune and inflammatory responses potentiate damage towards diverse parts of human body, substantial efforts have been spared to evaluate how inflammasome, either canonical or non-canonical, get involved in ADS’s etiology and pathogenesis in recent years. A significant body of literature has been published to indicate the bioactive beneficial effect of BAI, BAE, WGI and WGS at multiple organs and systems throughout the whole body. Since clinical features and molecular mechanism of ADS exert some certain differences, details on the ADS-ameliorative property of SBG’s small-molecular compounds will be described following the order of brain/spinal cord, skin/hair, gastrointestinal tract, joints, and others. Figure 2

### Brain and spinal cord

The best-known ADS which occur at brain and spinal cord include multiple sclerosis, narcolepsy and optical neuromyelitis. Multiple sclerosis is characterized as localized demyelination and constant overactivated neuroinflammation can be observed during the progress. Likewise, abnormally activated astrocytes mediate transduction of eIF-2α/ATF4/CHOP signaling and downstream elevated caspase-11 noncanonical inflammasome. LW-213, a derivative of WGI, was reported to modulate eIF-2α/ATF4/CHOP axis in human cutaneous T-cell (Yu et al., 2021), and given the fact that WGI could pass the blood brain barrier to regulate eIF-2α/ATF4/CHOP in neurons (Chen et al., 2015), it is likely to ameliorate multiple sclerosis via inhibiting caspase-11. Experimental autoimmune encephalomyelitis (EAE) is a recognized murine model of multiple sclerosis, several canonical inflammasome, namely NLRP3 (Ren et al., 2021), AIM2 (Ma et al., 2021), caspase-8 (Zhang et al., 2018) and NLRC4 (Biliktu et al., 2020), have been ascertained to play pivotal roles in EAE pathogenesis. Accordingly, BAI exerted therapeutic effect on the symptoms in EAE mice model and this effect was associated with its modulatory effect on STAT/NF-κB, which was significantly abolished after the pharmacological ablation of SOCS3 (Zhang Y. et al., 2015). Similar results were observed in a previous study conducted by Zeng and colleagues, in which EAE animal model was built in proteolipid protein 139–151 in SJL/J mice, and reduction of IL-4 and IFN-γ was observed following BAI administration (Zeng et al., 2007). Also, BAE showed potential to ameliorate EAE, and the underlying mechanism was associated with its 12/15-lipoxygenase inhibition, downstream microglia PPARβ induction as well as MAPK/NF-κB blockage (Xu et al., 2013). Similarly, cuprizone model is also an established murine model of multiple sclerosis. BAE reversed cuprizone-induced increase in Iba1-positive microglia, GFAP-positive astrocytes, as well as the gene expressions of CD11b, GFAP, TNFα, IL-1β, and iNOS to suppress neuroinflammation (Hashimoto et al., 2017; Sanadgol et al., 2017). It is also worth mentioning that scutellarin (STR) inhibited cuprizone-induced inflammatory response in neural stem cells, thereby rescuing behavioral deficits in this mouse model of multiple sclerosis, even STR is not one of the highest-content compounds from SBG (Wang et al., 2016).

Despite of the temporary shortage of direct evidence indicating BAE small-molecule compounds’ effect to suppressed the above four canonical inflammasome to ameliorate EAE, a range of current reports have demonstrated that they exerted potent negative effect on disease took place in central nervous system via suppressing canonical inflammasomes. For example, WGI mediated mitochondria dysfunction in malignant neuroblastoma cells via inducing activation of caspase-4 (Ge et al., 2015), the human ortholog of caspase-11 functioning as targeting the pathological hallmark of amyotrophic ateral sclerosis through a route of driving TDP-43 cytoplasmic accumulation in the primate brains. WGS also down-regulated NLRP3 inflammasome activation to alleviate traumatic spinal cord injury-induced inflammation (Zhu et al., 2017). To date, no report on SBG and its principal flavonoids’ effect on narcolepsy and optical neuromyelitis have been released.

### Skin and hair

Skin autoimmune diseases, such as psoriasis vulgaris, alopecia areata and systemic lupus erythematosus (SLE) affects approximately 200 million people globally. BAI and *ex vivo* expanded Foxp3+ regulatory T cells were recently reported to be promising therapeutics strategy for SLE treatment since it was able to reduce Tfh cell differentiation, IL-21 production and mTOR activation while promote the differentiation of Foxp3^+^ regulatory T cell (Yang et al., 2019). On the other hand, BAE demonstrated reno-protective effect in a mouse model of lupus nephritis, which is regarded as a representative manifestation in SLE. Authors also shed the light of the underlying mechanisms, indicating the ROS production, Nrf2/heme-oxygenase (HO)-1, NLRP3 inflammasome, as well as NF-κB phosphorylation are the dominant pathways regulated by BAE (Li D. et al., 2019). Of notes, whether SBG or its flavonoids have any affection on psoriasis vulgaris and alopecia areata remain to be clarified.

### Endocrine glands

Distinct from type 2 diabetes that caused by unhealthy lifestyles, T1DM is an undesirable consequence of autoimmune destruction (Syed, 2022). WGI demonstrated certain protective effect on diabetic renal injury, the mechanism involves its modulation on BCL-2-mediated autophagy and apoptosis and the inhibition of downstream pro-inflammatory cytokine production, which is likely to be associated with its inhibitory effect on osteopontin expression through enhancing PPARα activity (Zhang Y. M. et al., 2015; Liu et al., 2022). Besides, WGI’s ameliorative effect on STZ-induced symptoms like urinary albumin, histopathological damage and renal inflammation and fibrosis was related to its negative regulation on PI3K/Akt/NF-κB and TGF-β1/Smad3 (Zheng et al., 2020; Lei et al., 2021). Apart from T1DM secondary renal dysfunction, WGI was announced to alleviate diabetic cardiomyopathy through enhancing SOD1/2 and CAT while attenuating productions of ROS/MDA and proinflammatory cytokines (Khan et al., 2016).

Also in this model, BAI repaired T1DM-induced renal injury partly through modulating Klotho promoter methylation (a well-recognized endogenous inhibitor against renal fibrosis), as well as suppressing epithelial-to-mesenchymal transition (EMT) and microRNA-124/TLR4/NF-κB axis to repair renal fibrosis (Zhang S. et al., 2020; Zhang X. T. et al., 2020). Besides, several *in-vitro* studies verified the above results. For example, in TNF-α-stimulated pancreatic β-cell line Min6 which mimics β-cell destruction in TIDM, BAI reversed cell apoptosis and dysfunction via elevating miR-205 in a PI3K/AKT- and-NF-κB-dependent way (You et al., 2018). Endothelial dysfunction is commonly observed in T1DM. BAI was reported to alleviate endothelial impairment which was attributed to its role in reducing ROS via Akt/GSK3B/Fyn-mediated Nrf2 activation (Chen et al., 2018). A study conducted by Wang and colleagues revealed that cardiovascular system malformation induced by hyperglycemia was ameliorated by BAI administration due to its inhibition on ROS production and autophagy involving p62 ubiquitin, SOD, GSH-Px, MQAE and GABAA (Wang C. et al., 2018). Diabetic food ulcer is another complication of T1DM, the pathogenesis of which is proven to be correlated with elevated NO, MDA, p-ERK and p-HSP27 while decreased level of SOD, GSH, VEGF-c, Ang-1, Tie-2, TGF-β and Smad2/3. BAI administration reversed all the alterations and eventually promote the wound healing of food ulcer (Mao et al., 2021).

Accumulative evidence indicates BAE’s certain protective effect on T2DM (Li Y. et al., 2019; Wang Y. et al., 2022; Dong et al., 2022). The difference of T1DM and T2DM was firstly announced by Harold Percival Himsworth in 1936. ADS-associated T1DM patients completely lost their ability to produce insulin, while T2DM is a metabolic disease generally caused by insulin resistance and relative insulin deficiency, and is characterized by hyperglycemia (Ramirez et al., 2020; Lambrinoudaki et al., 2022). Of interest, current diabetic animal model seems to have no clear distinction between these two types, both of which were established by STZ injection. In brief, BAE, WGI and WGS prevented the progress of diabetic complications through regulating different pathways (Wang Y. et al., 2022). Mechanistically, for 1) Insulin resistance, BAE functioned as eliminating free radicals, restraining protein kinase C, enhancing α-Glucosidase activity and protecting β Cells, thus playing a role in reducing blood glucose, lipid and suppressing inflammatory reaction. WGI promoted GLUT4 protein level by activating PI3K-Akt pathway, and meanwhile facilitated glucose utilization via modulating PPAR-α pathway. WGS inactivated TLR4/NF-κB, NLRP3 and SOCS3to decrease IL-1 β, IL-6 and TNF-α secretion to trigger feedback regulation network. Aa for 2) Diabetic nephropaty, BAE rescued renal fibrosis via modulating AngII, TGF–β, α-SMA protein expression as well as p38MAPK/NF-κB transduction while WGS and WGI both negatively-regulated TLR4/NF-κB to protect renal tissue. Besides, for 3) Diabetic retinopathy, WGI restrained VEGF, bFGF, CTGF and TGF-β through ROS modulation, it also scavenged oxygen free radicals and reduced protein kinase C expression to potentiate vascular-protecting effect. Moreover, for 4) Diabetes peripheral neuropathy and cardiovascular diseases, BAE respectively regulated p38MAPK/Akt-Nrf2 pathway and AngII/SRP14 pathway to restore nerve conduction velocity and to repair endothelial cells peroxidation damage. Furthermore, WGI was reported to ameliorate hyperglycemia and hypoinsulinemia in T1DM mice through modulating p62DOK (Liu et al., 2018).

### Joints

Rheumatoid arthritis (RA) is an autoimmune disease that brings about constant chronic joint inflammation with no cure yet. In mice model of collagen-induced arthritis (CIA) which is widely used to mimic joint inflammatory symptoms in human RA (Wu et al., 2022), BAI treatment alleviated radiographic and histologic abnormalities in the hind paw joints of CIA rats, and the underlying mechanism was associated with its modulation on TLR2/MYD88/NF-κB p65 signaling as well as the blockage of IL-1β, TNF-α and IL-6 production (Wang H. Z. et al., 2014; Xu et al., 2018; Bai et al., 2020). BAI also lowered relevant proinflammatory cytokines including TNF-α, IL-1β, IL-6, MMP-2, MMP-9, NO and COX-2 secretions in e synovial fluids and tissues of CIA rats to interfere JAK/STAT signaling transduction, which attributed to its restoring of pressure pain thresholds and clinical arthritis scores (Wang G. et al., 2018). In addition to collagen II, RA animal model established by complete Freund’s adjuvant (CFA) is also well-recognized because it shares similar experimental designs, immunological and pathological characteristics with RA in human beings (Huang et al., 2020). In this model, WGI reduced arthritic score and paw thickness-mediated by CFA and decreased IL-1β, IL-6, and TNF-α production via blocking MAPK and NF-κB signaling (Huang et al., 2020). Of notes, ICAM-1 was reported to occur in RA and meanwhile IL-17 are abundantly expressed in synovial fibroblasts. Since IL-17 not only mediates the synthesis of ICAM-1 and diverse cytokines (IL-6 and TNF-α, etc), but also accelerates collagen degradation via diminishing collagen and proteo glycan synthesis while enhancing bone erosion, its inhibition was believed to be an effective way to ameliorate RA. BAI rescued ankle swelling in Rats model of RA through negatively-regulating Th17 cell population expansion in spleen, and this effect was exerted through reducing the gene expression of RORgt, a dominant transcription factor in Th17 cell differentiation (Dinda et al., 2017; Sun and Gu, 2019).

### Autoimmune uveitis

Targeting immunological inflammation has been regarded as a promising way to prevent pathogenesis of experimental autoimmune uveitis (EAU), a sight-threatening ocular inflammatory disorder. Zhu and colleagues declared BAI demonstrated protective effect on EAU due to its inhibition on intraocular inflammatory process, as well as infiltrated inflammatory cells and transcriptions of proinflammatory cytokines like IL-17A, IFN-γ and TNF-α. Moreover, it is observed that BAI promoted regulatory T cells’ amounts and frequency while restrained those of Th1 and Th17. Similar results were obtained in *in-vitro* study which further drew a conclusion that BAI was a promising candidate for EAU treatment since it modulated reg/Teff balance as a inducer of aryl hydrocarbon receptor (Zhu et al., 2018). Besides, the pathogenesis of EAU could also be rescued by BAE administration with an underlying mechanism of IL-17 inhibition (Li et al., 2016). Research concerning the interaction between WGI, WGS, or SBG and EAU is yet limited.

### Digestive system

Comprised with ulcerative colitis and Crohn’s disease, inflammatory bowel disease (IBD) is characterized as intestinal microbiota alterations which are linked with a wide range of autoimmune conditions (Spalinger et al., 2022). A recent released review concentrated on the up-to-date studies investigating the affection of baicalin on IBD, indicating BAI can be a promising therapeutic prospect as a potential supplementary agent due to it reversed IBD by virtue of anti-inflammation and antioxidant properties, immune regulation, as well as maintenance of intestinal barrier and intestinal flora balance (Wang X. et al., 2022). Liang and colleagues found that BAI, BAE, and the combination of the two flavonoids (those extracted either young or withered SBG) blocked NF-κB and MAPK signaling to alleviate clinical symptoms and signs of IBD with different potency, they also repaired the abnormal organ indices and normalized the blood biochemistry (Liang et al., 2019). Besides, a novel agent named PF2405 which enriched with BAE, oroxylin A and WGI had outstanding preventive and therapeutic activities in trinitrobenzene sulfonic acid or dextran sulfate sodium-induce colitis mice. Mechanically, PF2405 relieved the histopathological severity and meanwhile decreased the expression of COX-2, TNF-α, and IL-1β in colon tissue, and this effect was correlated with its abrogating to inflammatory response mediated by COX-2/MAPK signaling (Jiang et al., 2015). A study conducted by Zhu and colleagues shed the light of BAI ameliorated DSS-induced IBD via regulating macrophage polarization to the M2 phenotype. In specific, it dampens LPS-induced tumor necrosis factor α, IL-23 and IRF5 expression while enhancing IL-10, arginase-1 (Arg-1) and IRF4 expression in colon, to reverse macrophage subset redistribution (Zhu et al., 2016). Of notes, a range of ancient prescriptions that contains SBG also demonstrated certain ameliorative effect on IBD in addition to small-molecules from SBG, typical ones include Jiawei Gegen Qinlian decoction (Li et al., 2021), Banxia Xiexin decoction (Wang et al., 2021), Gegen Qinlian decoction (Xu et al., 2021) and Huangqin decoction (Chen et al., 2016), among which BAE, WGI, and BAI are the principal contents accounting for these bioactive effects, and the principal involved molecular pathways include NF-κB, NLRP3 inflammasome, MAPK, JAK/STAT3, IL-17, the Th17 cell differentiation and oxidative stress pathway.

Autoimmune hepatitis (AIH) is another autoimmune disease occurring in digestive system. Abnormal apoptosis in activated lymphocytes accelerates the development of AIH. In AIH animal model established by concanavalin A, BAE was observed to reduce Bcl-2/Bax ration and meanwhile restraining caspase-9/-3 activation to further induce apoptosis in activated lymphocytes and eventually ameliorate AIH (Zhang et al., 2013). Of interest, SBG extract itself was reported to be a AIH model inducer when intraperitoneally-administrated to mice (Wang J. Y. et al., 2014). The reason for these controversial results may be related to the potential opposite effect of SBG constituents on AIH. Given the fact that reports on how AIH and constituents affect AIH remain limited, further studies concerning the effect of BAI, WGI and WGS on AIH are desperate to figure out the underlying mechanisms.

## Discussion

Due to the limitation of current therapeutic strategies which remain simplistic and reductionist understanding of ADS pathogenesis, it is urgent to explore novel alternative inhibitors with natural sources. In clinical, considerable chemical-modified agents show straightforward ameliorative effect on ADS by relieving symptoms broad-actively, while not disease-specifically (Wu et al., 2021). Of notes, adverse reactions such as infusion reactions, human anti-chimeric antibodies production, and neutropenia cannot be avoided in ADS-relieving approaches. Meanwhile, combining similarities among distinct ADS indeed brings about a grey zone for diagnosis by young clinicians since not all ADS share similar symptoms (Wu et al., 2018). The new generation of biological drugs emerged in past decade are targeted more efficient, more specific, and safer, which provides better options for ADS treatment. The representative ones include inhibitors targeting JAK and some species of monoclonal antibodies. However, those biological agents as well increase the financial burden for ADS patients (Tavakolpour, 2017). Thus, the current therapeutic regimen’s inefficacy in certain cases coupled with low rates of success as well as numerous side effects, which do open new avenues for the role of natural products as novel therapeutic agents for auto-inflammatory disorders. SBG is one of the most important and high-frequently used traditional Chinese medicine where dried roots preparation of ‘Huang-qin’, as well as bioactive constituents, particularly of those flavonoids, are applied for liver and pulmonary complaints and as alternative and complementary treatment for various of carcinoma, such as prostate, bladder, breast, and liver cancer (Bonham et al., 2005; Zhao Q. et al., 2019; Cao et al, 2020; Kong et al., 2021; Yao et al., 2021). However, no particular attention concerning how SBG affect ADS in a systematic way has been paid by far. By combing through the current reported evidence, even the impact of SBG on ADS symptoms is also broad-active like chemical-modified drugs, it has characteristic of multi-component and multi-targeting which potentiates mutual-reinforcement at both pharmacokinetic and pharmacodynamics level as displayed in Figure 2 and Table 2, and this goal is achieved through 1) superimposing potency on one certain target, 2) expanding the scope of efficacy or 3) promoting the intracellular transport of SBG flavonoids via affecting membrane transport proteins like P-gp and organic cation transporters (Wu et al., 2016). Compared with the chemical-modified agents currently used in clinic to reverse ADS, SBG oral preparation and its bioactive ingredients have advantage of relatively low toxicity of only causing symptoms such as stomach discomfort and muscle soreness, which suggest that it a promising candidate to be widely applied in clinical practices (Zhao T. et al., 2019). Indistinct diagnosis of different types of ADS has been being an enormous challenge for young clinicians. To cope with the diverse symptoms, patients may receive drug combination which is likely to lead to the accumulation of adverse reactions. As other traditional Chinese medicine, the characteristic of SBG is multi-component and multi-targeting which makes it possible to avoid toxicity superposition since no obvious adverse reaction is observed in oral preparation of SBG (Zhao Q. et al., 2019). In fact, SGB has a wide distribution in many provinces of China including Heilongjiang, Liaoning, Inner Mongolia, Hebei, Henan, Gansu, Shanxi, Shandong, and Sichuan, etc. Besides, it is also abundant in other countries like Soviet Union in Eastern Siberia, Mongolia, North Korea, and Japan. It is noteworthy that SBG grows on sunny grassy slopes as well as desert lands at an altitude of 60–2000 m, the nature of easily-accessible allows it to be finanial-friendly to ADS patients.

**TABLE 2:** JAK/STAT/mTOR, BAX/Bcl2/caspase-3/9, TLR4/NF-κ/PPAR, IL-4/PI3K/Akt/GSK3β, TGF-β/Smad2/3,NLRP3/caspase-1/GSDMD and ROS/Nrf2/HO-1 pathways are the principal signaling modulated by SBG and its compounds in ADS. The reported molecules regulated by SBG are marked with a red star.JAK/STAT/mTOR, BAX/Bcl2/caspase-3/9, TLR4/NF-κB/PPAR, IL-4/PI3K/Akt/GSK3β, TGF-β/Smad2/3,NLRP3/caspase-1/GSDMD and ROS/Nrf2/HO-1 pathways are the principal signaling modulated by SBG and its compounds in ADS. The reported molecules regulated by SBG are marked with a red star.

Compounds	ADS	Models	Dose	Effect	References
BAI	SLE	Lupus-prone MRL/lpr mice	200 mg/kg, i.p	Tfh cell differentiation and IL-21 production ↓, Foxp3^+^ regulatory T cell differentiation ↑, mTOR ↓	Yang et al. (2019)
	EAE	MOG _35–55_- challenged C57BL/6 mice	100 mg/kg, i.p	IL-17, IFN-γ, GM-CSF, IL-10, IL-5 and IL-4 ↓, p-STAT1, p-STAT3 and p-STAT4↓, Th1/Th17 capacity↓ SOCS induction ↑	Zhang et al. (2015a)
		proteolipid protein (PLP) _139–151_-stimulated SJL/J mice	5–10 mg/kg, i.p	Clinical score ↓, histological score ↓	Zeng et al. (2007)
		PLP-stimulated popliteal and inguinal lymph node mononuclear cells from EAE mice	5–25 μM	IFN-γ ↓, IL-4 ↑	
	T1DM	STZ-challenged Kunming mice	40 mg/kg, po	Renal injury ↓, CD3, CD68, p65 and iκBα ↓, Nrf2 ↑, E-Cadherin, ɑ-SMA, β-catenin, TGF-β1 and p-smad2/3 ↓, MMP13↑, Klotho promoter hypermethylation ↓	Zhang et al. (2020a)
		High glucose-stimulated HK2 cells	--	Klotho ↑, Dnmt1, Dnmt3a, and Dnmt3b ↓	
		TNF-α-stimulated pancreatic β-cell Min6	25–50 μM	cell apoptosis ↓, miR-205 ↑, PI3K/AKT and NF-κB pathway ↓	You et al. (2018)
		STZ-challenged SD rats	25–100 mg/kg, po	NO, MDA, p-ERK and p-HSP27 ↓, SOD, GSH, VEGF-c, Ang-1, Tie-2, TGF-β and Smad2/3 ↑	Mao et al. (2021)
		STZ-challenged C57BL/6 mice	15–45 mg/kg, po	Fibronectin and collagen IV↓, miR124 ↑	Zhang et al. (2020b)
		High glucose-stimulated HK2 cells	100 μM	Fibronectin and collagen IV↓, miR124 ↑ TLR-4/NF-κB ↓	
		STZ-challenged C57BL/6 mice	50 mg/kg, i.p	endothelial dysfunction ↓ BAX/Bcl-2 ↓, c-caspase-3 ↓	Chen et al. (2018)
		High glucose-stimulated HUVECs	50 μM	Akt and GSK3B phosphorylation↑, nuclear export of Fyn↑, nuclear localization of Nrf2 ↑	
		Hyperglycemia-exposed early chick embryos	6 μM	ROS production ↓, autophagy ↓	Wang et al. (2018a)
	RA	collagen II monoclonal antibody-elicited Balb/c mice	30 mg/kg, i.p	pressure pain thresholds and clinical arthritis scores ↓ TNF-α↓, IL-1β ↓, IL-6↓, MMP-2↓, MMP-9↓, iNOS↓, COX-2↓, JAK1/STAT3↓	Wang et al. (2018b)
		collagen II-challenged SD rats	15–60 mg/kg, po	TNF-α↓, IL-1β ↓, IL-6↓, TLR2, MyD88 and NF-κBp65 ↓	Bai et al. (2020)
		collagen II-challenged Wistar rats	50–100 mg/kg, i.p	TNF-α↓, IL-1β ↓, NF-κBp65 acetylation ↓, sirt1↓	Wang et al. (2014a)
		collagen II-challenged C57BL/6 mice	20 mg/kg	Arthritis scores ↓, Paw swelling↓	Xu et al. (2018)
		collagen II-challenged DBA/1 mice	200 mg/kg, i.p	Arthritis severity↓, TNF-α↓, IL-6↓, IL-17↓, IL-1β↓, p-JAK2 and p-STAT3 ↓, T cell percentage ↓	Sun and Gu, (2019)
		CFA-challenged C57BL/6 mice	100 mg/kg, i.p	ankle swelling↓, Th17 cell population expansion in spleen↓, RORgt gene expression↓	Dinda et al. (2017)
	IBD	DSS-challenged C57BL/6 mice	20 mg/kg, i.p	Colon length ↑, Histology score↓, IFN-γ ↓, IL-6 ↓, IL-17↓	Xu et al. (2018)
		epithelial cell	200 μM	STAT4 ↑	
	IBD	high-sugar and high-fat diet, a high temperature and humidity environment (HTHE), excess drinking, and infection of *Escherichia coli*.	100 mg/kg, po	clinical symptoms ↓, IL-6, IL-1β, and IL-17↓, NF-κBp65↓, p38MAPK↓, STAT3 ↓	Liang et al. (2019)
	EAU	hIRBP1-20 and H37Ra in CFA-challenged C57BL/6 mice	0–200 mg/kg, i.p	EAU symptoms and pathological manifestation ↓	Zhu et al. (2018)
				IFN-γ, IL-17A, and TNF-α↓, IL-10 ↑, Tregs ↑, Teffs ↓, AhR ↑	
BAE	EAE	MOG _35–55_- challenged C57BL/6 mice	100 mg/kg, i.p	EAE severity↓ mRNA of CCL2, CCL3, CCL20, CXCL10, IFN-γ and IL-17↓	Xu et al. (2013)
		LPS-stimulated primary microglia, astrocytes and BV2 cells	10–20 μM	mRNA of CCL2, CCL3, CCL20, CXCL10, TNFα, IL-1β, IL-6 and IL-12p40↓, 12/15-lipoxygenase ↓, PPAR ↑	
	SLE	Pristane-challenged Balb/c mice	25–100 mg/kg, i.p	IL-1β ↓, IL-18↓, ROS↓, NLRP3 inflammasome↓, p-NF-κB ↓, Nrf2↑, HO-1↑	Li et al. (2019a)
		LPS + ATP-stimulated myeloid-derived suppressor cells	0.01–0.04 μM	IL-1β ↓, IL-18↓, ROS↓, NLRP3 inflammasome↓, p-NF-κB ↓, Nrf2↑, HO-1↑	
	T1DM	STZ-challenged Wistar rats	2–4 mg/kg, i.p	Memory function ↑, ChAT activity↑, blood glucose ↓, AChE activity↓, p-PI3K↑, p-Akt ↑, p-GSK3β↓, caspase-9 ↓ and caspase-3↓	Qi et al. (2015)
		STZ-challenged Wistar rats	100 mg/kg, po	Blood pressure ↓, AGEs ↓, TNFα↓, NF-κB ↓and inhibited histopathological changes ↓	El-Bassossy et al. (2014)
		STZ-challenged SD rats	150 mg/kg, po	microglial activation ↓, TNF-α, IL-18, IL-1β↓, GFAP and VEGF expression from Mu¨ller cells↓, vascular abnormality and ganglion cell loss within the retina ↓	Yang et al. (2019)
	RA	collagen II-challenged C57BL/6 mice	20 mg/kg	Arthritis scores ↓, Paw swelling↓, IFN-γ ↓, TNF-α↓, IL-2 ↓, IL-17↓	Xu et al. (2018)
		epithelial cell	200 μM	STAT3↓, STAT4↓	
	EAU	R14 in CFA-induced Lewis rats	2.5 mg/kg, i.p	IL-17 ↓	Li et al. (2016)
	IBD	high-sugar and high-fat diet, a high temperature and humidity environment (HTHE), excess drinking, and infection of *Escherichia coli*.	100 mg/kg, po	clinical symptoms ↓, IL-6, IL-1β, and IL-17↓, NF-κBp65↓, p38MAPK↓, STAT3 ↓	Liang et al. (2019)
		DSS/TNBS-challenged C57BL/6 mice	25 mg/kg, po	Colon length ↑, TNF-α ↓, IL-1β ↓, COX-2 ↓	Jiang et al. (2015)
		TNF-α-stimulated HT-29 cells	0–50 μg/ml	COX-2 ↓, p-ERK1/2↓, p-p38↓, p-JNK↓	
	AIH	Con A-challenged C57BL/6 mice	100 mg/kg, i.p	cytochrome c ↑, caspase-9↑, caspase-3↑, ALT↓, IFN-γ ↓, TNF-α↓	Zhang et al. (2013)
		Con A-activated CD3^+^ T cells	0–20 μM	caspase-9↑, caspase-3↑, caspase-8↑, BAX/Bcl-2 ↓	
WGI	T1DM	STZ-challenged C57BL/6 mice	10–40 mg/kg, po	NF-κB ↓, podocyte apoptosis↓, podocyte autophagy ↑	Liu et al. (2022)
		High glucose-stimulated MPC5 cells	4–16 μM	TNF-α, MCP-1, IL-1β and p-p65 ↓	
		STZ-challenged C57 mice	10 mg/kg, i.p	Osteopontin ↓	Zhang et al. (2015b)
		3T3-L1 adipocytes	0–20 μM	Osteopontin ↓, p38MAPK↓, PPARα↑	
		STZ-challenged C57BL/6 mice	10–40 mg/kg, po	urinary albumin and histopathological damage in tubulointerstitium ↓, PI3K/Akt/NF-κB ↓	Lei et al. (2021)
		High glucose-stimulated HK2 cells	4–16 μM	PI3K/Akt/NF-κB ↓	
		STZ-challenged C57BL/6 mice	10–40 mg/kg, po	albuminuria and histopathological lesions ↓, TNF-α, MCP-1, IL-1β↓, fibronectin, collagen IV, α-SMA, and TGF-β1 ↓	Zheng et al. (2020)
		High glucose-stimulated glomerular mesangial cells SV40	1.5825–6.25 μg/ml	TNF-α, MCP-1, IL-1β↓, fibronectin, collagen IV, α-SMA, and TGF-β1 ↓	
		STZ-challenged C57BL/6 mice	10 mg/kg, i.p	IL-1β, IL-6, TNFα, and PAI-1 ↓, SOD1/2 and CAT ↑, ROS/MDA production↓	Khan et al. (2016)
		High glucose-stimulated primary neonatal rat ventricular myocytes	10 μM	IL-1β, IL-6, TNFα, and PAI-1 ↓, SOD1/2 and CAT ↑, ROS/MDA production↓	
	RA	CFA-challenged Wistar rats	25–50 mg kg, po	Arthritic score↓, paw thickness ↓, MAPK and NF-κB↓, IL-1β, IL-6, and TNF-α↓	Huang et al. (2020)

In fact, up to 40 constituents have been isolated from SBG, among which BAI, BAE, WGS, and WGI are used as quality control indicators for SBG and its related preparations, and these four flavonoids are accounted for SBG’s bioactive properties and the key players in SBG’s clinical therapeutic effects (Wang Y. et al., 2022). To date, there has been no large-scale investigation of the actual clinical use of SBG, not only the distribution but also the frequency of use. Liao and colleagues traced an available robust healthcare system with complete database records, indicating SBG was recommended to combat a range of chronic diseases including respiratory inflammatory disorders, headache, sleep impairments, hepatitis, diarrhoea, vomiting, haemorrhage, hypertension, and gastrointestinal discomfort (Liao et al., 2021), These clinical applications mentioned above can dramatically be linked to the modern concept of anti-infection or anti-inflammation, which is specified in the Chinese Pharmacopoeia, paving the development of SBG as a natural anti-inflammatory, antibiotic, and anti-tumor drug (Chen et al., 2018). For the flavonoids of SBG, BAI has anti-microbial activity by exerting potent synergistic effect with penicillin G/amoxicillin against 20 clinical penicillinase-producing S. aureus strains. Meanwhile, the clinical trial of WGI as an anti-cancer agent candidate has recently been approved by the State Drug Administration of China (Wang Z. L. et al., 2018). However, clinical studies concerning whether and how SBG affect ADS are warranted to confirm the possible beneficial therapeutic outcome and the underlying mechanisms.

**FIGURE 3:**
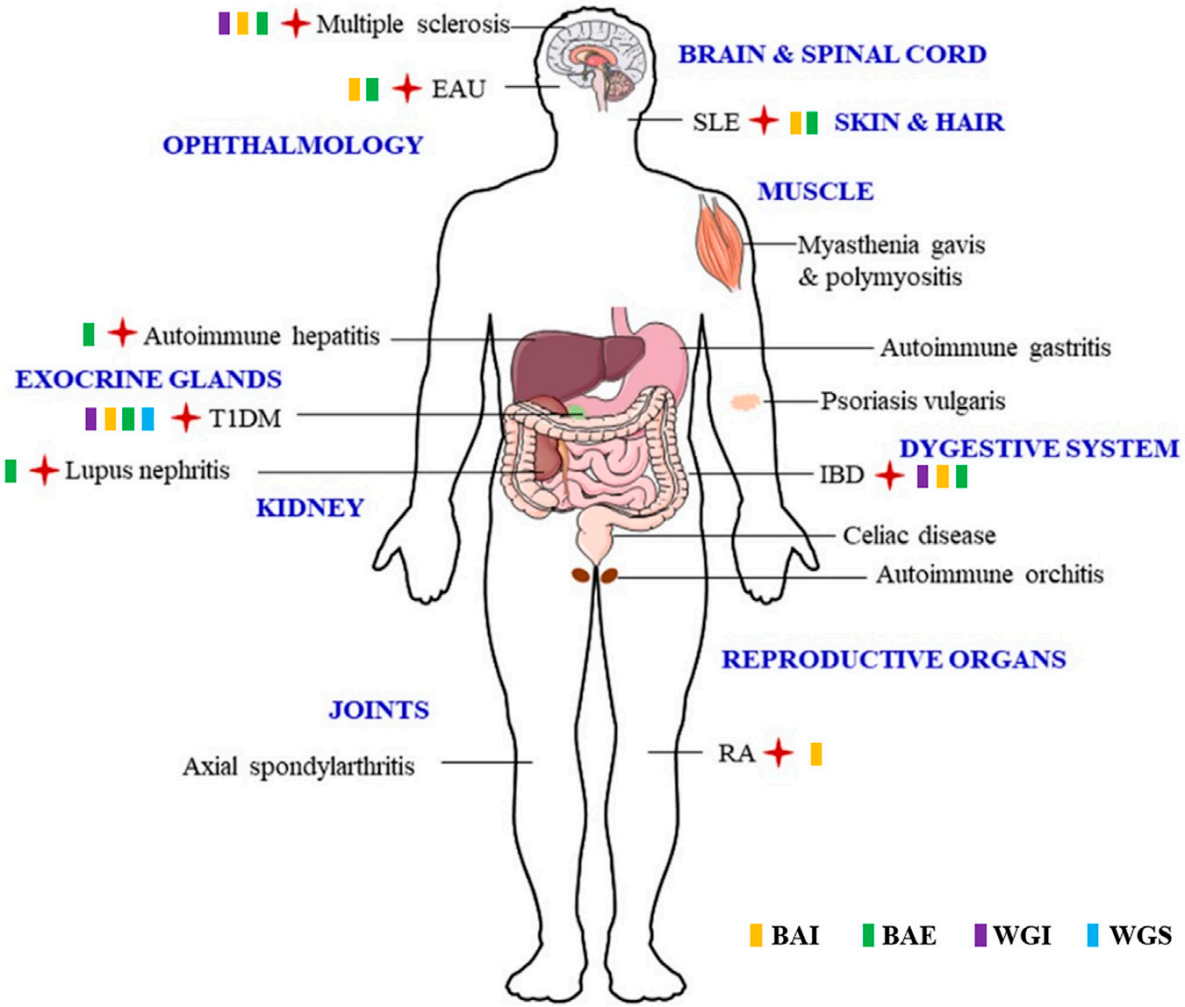
The verified ADS affected by SBG and its flavonoids are highlighted by red cross, and the effective flavonoid is marked by relevant symbols.

Although the issue of bioavailability is always being questioned for SBG’s flavonoids or even the majorities of small-molecular compounds with natural resource, SBG and its flavonoids were reported to have ameliorative effect on disorders occurred in multiple systems, thereby suggesting those flavonoids were able to reach targeted tissues via bloodstream and crossing the blood-brain barrier to perform therapeutic effect on ADS took place in diverse systems illustrated in Figure 3. The commercial interest and the increasing demand of SBG contributed to the appearance of huge gap of its development. Given the evidence provided by available scientific validation, SBG and its principal flavonoids, namely BAI, BAE, WGI and WGS, are potential candidates since they block abnormal auto-inflammatory response which occurred in a wide range of ADS including multiple sclerosis, EAU, SLE, T1DM, RA, IBD, as well as AIH. As discussed in previous chapters in this review, SBG and its principal flavonoids modulates diverse mechanistic routes to maintain their inflammatory-preventing and immune-regulatory actions. Notably, pathways of JAK/STAT/mTOR, BAX/Bcl2/caspase-3/9, TLR4/NF-κB/PPAR, IL-4/PI3K/Akt/GSK3β, TGF-β/Smad2/3, NLRP3/caspase-1/GSDMD and ROS/Nrf2/HO-1 are the key ones modulated by SBG. Nevertheless, there is still a long way before fully uncover the underlying molecular of SBG ameliorates ADS. In recent years, an increasing body of evidence ascertained canonical and noncanonical inflammasome activation pave the way for the development and exacerbation of ADS. During the process of ADS pathogenesis, immune tolerance dysfunction is believed to bring about unexpected pro-inflammatory debris accumulation and gargantuan cytokines and chemokines release, which further fuels cytokine storm and cause the secondary damage to tissues and organs (Fajgenbaum and June, 2020). The certain involvement of inflammasome in ADS progress has been revealed in the past decades while there are still many underlying mechanisms remaining to be clarified. Generally, inflammasomes can be divided into canonical ones (such as NLRP3, AIM2 and NLRP1, etc) and noncanonical caspase-11. The former category is architecturally assembled by a sensor (also known as recognition receptor), an adaptor and a protease effector in response to a wide range of exogenous and endogenous stimuli. Noncanonical caspase-11 inflammasome activation, on the other hand, directly triggered by LPS that gains access to cytosol with a dispensable role played by TLR-4 (Abu Khweek and Amer, 2020; De Carvalho Ribeiro and Szabo, 2022). Numerous evidence emerged in recent years indicate that canonical and noncanonical inflammasomes are of equal importance when induing pyroptotic cell death, the consequence of which is one of the principal reasons accounting for ADS exacerbation (Wu et al., 2021). Herein, targeting either canonical or noncanonical inflammasome thereby is an attracting way to calm down cytokine storm thereby preventing development and exacerbation of ADS. Unfortunately, studies concerning whether and how SBG and its flavonoids affect inflammsomes/pyroptosis pathways are still limited, future investigations are encouraged to focus on the interactions between SBG and various kind of inflammasomes such as AIM2, NLRP1 and caspase-11. In addition, Neutrophil extracellular traps (NETs), the fibrous networks which protrude from the membranes of activated neutrophils was reported to play a central role in ADS pathogenesis (Lee et al., 2017), it will be of interest to ascertain whether NETs is a target of SBG in future studies.

In almost all ADS cases, there is a definite sex difference in prevalence, whereby females are generally more frequently affected than that of males (almost three more times), and organ vulnerability as well as reproductive capacity are most vital factors facilitating this distinction (Hodson, 2021; Ngo et al., 2014). However, the current studies concerned more about the molecular mechanism while hardly conducted experiments to compared the sex difference in the therapeutic effect of SBG on ADS. Further investigations may be needed to address such questions.

## Conclusion

To sum up, to introduce a new generation of treating regimen for ADs with fewer life-threatening side effect, it is desperate to explore novel small-molecule alternative medicine from natural resources. With advantages of multi-targeting and broadly-active with low adverse reactions and less financial burden, SBG and its flavonoids are promising candidates for the treatment of diverse ADS including multiple sclerosis, systemic lupus erythematosus, type 2 diabetes and the complications, rheumatoid arthritis, autoimmune uveitis, inflammatory bowel disease, and autoimmune hepatitis. Nevertheless, bioavailability issue need resolving and detailed information on the underlying molecular mechanism and gender difference in this process need to be addressed and supplemented before SBG and its flavonoids can be translated from bench to bedside.
